# Cryptic species obscure introduction pathway of the blue Caribbean sponge (*Haliclona* (*Soestella*) *caerulea*), (order: Haplosclerida) to Palmyra Atoll, Central Pacific

**DOI:** 10.7717/peerj.1170

**Published:** 2015-08-06

**Authors:** Ingrid S. Knapp, Zac H. Forsman, Gareth J. Williams, Robert J. Toonen, James J. Bell

**Affiliations:** 1 School of Ocean and Earth Science and Technology, Hawaiʻi Institute of Marine Biology, University of Hawaiʻi at Manoa, Kāneʻohe, HI, USA; 2 Center for Marine Biodiversity and Conservation, Scripps Institution of Oceanography, La Jolla, CA, USA; 3 School of Biological Sciences, Victoria University of Wellington, Wellington, New Zealand

**Keywords:** Phylogeography, Spicules, Caribbean, Atoll lagoon, Spicules, Micromorphology, mDNA, nDNA, Non-indigenous species, Porifera

## Abstract

Cryptic species are widespread across the phylum Porifera making the identification of non-indigenous species difficult, an issue not easily resolved by the use of morphological characteristics. The widespread order Haplosclerida is a prime example due to limited and plastic morphological features. Here, we study the reported introduction of *Haliclona* (*Soestella*) *caerulea* from the Caribbean to Palmyra Atoll via Hawaiʻi using morphological characteristics and genetic analyses based on one nuclear (18s rDNA) and three mitochondrial (COI, the barcoding COI extension (COI ext.) and rnl rDNA) markers. Despite no clear division in lengths of the oxea spicules between the samples, both mtDNA and nDNA phylogenetic trees supported similar topologies resolving two distinct clades. Across the two clades, the concatenated mtDNA tree resolved twelve subclades, with the COI ext. yielding most of the variability between the samples. Low sequence divergence values (0.68%) between two of the subclades indicate that the same species is likely to occur at Palmyra, Hawaiʻi and the Caribbean, supporting the hypothesis that *H. caerulea* was introduced to Palmyra from the Caribbean, although whether species came directly from the Caribbean to Palmyra or from Hawaiʻi remains unresolved. Conversely, the pattern of highly divergent cryptic species supports the notion that traditionally used spicule measurements are taxonomically unreliable in this group. This study illustrates how understanding the scale of within- as opposed to between-species level genetic variation is critical for interpreting biogeographic patterns and inferring the origins of introduced organisms.

## Introduction

The identification of a species is a basic, yet fundamental part of conservation and management but is complicated by the lack of distinct morphological traits in many marine taxa (Bickford et al., 2007; Forsman et al., 2010). This problem is particularly apparent in the phylum Porifera (sponges) (Grant, 1836), which can often be difficult to identify in the field due to their limited, but plastic morphologies in response to environmental variability (Palumbi, 1984; Wulff, 2001; Bell, Barnes & Turner, 2002; McDonald, Hooper & McGuinness, 2002).

There are 8,500 known sponge species, with an estimated 15,000 in total (Hooper & Van Soest, 2002; Van Soest et al., 2012). Despite their great diversity, all functional sponges perform similar but key roles in ecological systems (Bell, 2008). They are efficient spatial competitors (Bell & Barnes, 2003; Wulff, 2006) frequently possess chemical defences to deter predators (Schwartz, Luikart & Waples, 2007) and remove organic and inorganic particles and nutrients (Gili & Coma, 1998), which can greatly impact water column characteristics (Fu et al., 2006; Peterson et al., 2006; Fu et al., 2007; De Goeij et al., 2013). All these factors combine to pose a potential threat to ecosystems as invasive species (Coles et al., 2007). While the early detection and eradication of introduced species is the best strategy when prevention fails (Mehbub et al., 2014), there is a trade-off between early detection and action, and sufficient information for informed management decisions, especially in the case of cryptogenic species, where the species origins are unknown. For example, apparently well-documented introductions of an alien invasive coral (*Carijoa* spp.) and seahorses (*Hippocampus* spp.) in Hawaiʻi have proven false when examined genetically (Concepcion et al., 2010; Szabó et al., 2011). Misidentification of cryptic species can also have dramatic impacts on conservation (Bowen, Nelson & Avise, 1993; Ravaoarimanana et al., 2004). Therefore, it is also important to determine the identification of introduced species, especially taxonomically difficult and cryptogenic taxa such as sponges, before substantial management resources are wasted on misinformation.

As with most past taxonomic work, classification and species description for sponges has been based on morphological characteristics such as spicules and spongin architecture (Hooper & Van Soest, 2002). However, morphological plasticity and the low number of phenotypic characters in sponges can make consistent and accurate identification problematic (Wulff, 2001; Wörheide & Erpenbeck, 2007). For example, some sponge species that have been long believed to be cosmopolitan, spanning large geographic ranges, have now been revealed, using molecular markers, as complexes of cryptic species (Klautau et al., 1999; Xavier et al., 2010; Swierts et al., 2013; Bell et al., 2014). Therefore, genetic markers have become important tools to identify divergent cryptic species and have revealed that the distribution ranges of many so-called “cosmopolitan species” have often been overestimated because they are actually not a single species (Solé-Cava & Thorpe, 1987; Boury-Esnault, Solé-Cava & Thorpe, 1992; Klautau et al., 1999; Hoshino, Saito & Fujita, 2008). Confirming the identity of an introduced species is therefore the first step to studying its phylogeographic distribution and introduction pathway (e.g., Concepcion et al., 2010).

Within the phylum Porifera lies the class Demospongiae (Sollas, 1885), which encompasses approximately 83% of the known sponge species making it the largest class in the phylum Porifera (Van Soest et al., 2012). The order Haplosclerida (Topsent, 1928) is one of the most speciose and diverse orders in this class and includes the majority of freshwater species and a large number of shallow-water marine sponges (Van Soest & Hooper, 2002), although the monophyly of this class remains contentious (Redmond et al., 2011; Cárdenas, Pérez & Boury-Esnault, 2012; Redmond et al., 2013). The range of spicule types in Haplosclerida is very limited—megascleres are either oxeas or strongyloxeas and the microscleres, if present, are sigmas, microxeas, microstrongyles or toxas—making classification based on morphological characters difficult compared to sponge groups such as Tetractinellida (Marshall, 1876) and Calcispongia (Schmidt, 1862), which have more distinct morphological features (Chombard, Boury-Esnault & Tillier, 1998; Manuel et al., 2003). More recently, genetic analyses using a range of markers have been employed to resolve species relationships within the Haplosclerida, which is currently divided into 2 to 4 suborders; however, there are still major incongruences among molecular markers as to how many suborders exist and their relationships (McCormack, Erpenbeck & Van Soest, 2002; Itskovich et al., 2007; Redmond et al., 2011). At the species level, there have also been a number of examples of taxonomic confusion. For example, in the genus *Haliclona* (Grant, 1836), where multiple samples of the same species; *H. oculata* (Pallas, 1766) and *H. cinerea* (Grant, 1826), were collected, both had individuals (identified as the same morphospecies) that were subsequently found to be genetically distinct and distantly related according to both mitochondrial CO1 and 18s rDNA markers (Raleigh et al., 2007; Redmond & McCormack, 2008). Such taxonomic challenges have resulted in a lack of resolution at all levels of the Haplosclerida order, which complicates many ecological, evolutionary, and phylogeographic studies (Raleigh et al., 2007).

The aim of this study was to investigate the reported introduction of *Haliclona (Soestella) caerulea* (Hechtel, 1965) (order Haplosclerida) to the Central Pacific from the Caribbean via Hawaiʻi (DeFelice, Eldredge & Carlton, 2001; Coles et al., 2006; Knapp et al., 2011). It is thought that *H. caerulea* was introduced first to Hawaiʻi in the second half of the last century as it was not found by sponge taxonomists during surveys in the 1950’s (De Laubenfels, 1950b) or 1960’s (Bergquist, 1967) where it is now abundant (DeFelice, Eldredge & Carlton, 2001; Coles et al., 2006). It was then believed to have been transported from Hawaiʻi to Palmyra Atoll, a near-pristine oceanic atoll in the Central Pacific, via fouling on ships, yachts and barges (Godwin, 2003; Knapp et al., 2011) or other structures e.g., floating dry-docks (Godwin & Eldredge, 2001). Ship fouling is believed to be the primary pathway of introduction of alien species to Hawaiʻi (Molnar et al., 2008), and Hawaiʻi has long been the predominant shipping connection to Palmyra (Dawson, 1959; Knapp et al., 2011). The limited larval dispersal capabilities of *H. caerulea* (to several meters) (Maldonado & Young, 1999) also suggests that the chances of this species naturally increasing its range from the Caribbean to the Central Pacific is highly unlikely. However, considering the limited micromorphological variability within Haplosclerida species (Redmond et al., 2011) and considerable taxonomic uncertainty within the order, it is likely that there is undetected biodiversity (cryptic species) or misidentifications. Thus, it is also possible that sponges with a similar appearance in the lagoons at Palmyra, which are believed to be introduced, may prove to be endemic because of taxonomic confusion within the group. Confirming whether the Caribbean species *H. caerulea* was introduced to Palmyra Atoll via Hawaiʻi may be important for future management of both locations by establishing the possible paths of introduction as well as aiding in the future monitoring of this species distribution (Schwartz, Luikart & Waples, 2007). In this study, spicule (oxea) length measurements of *H. caerulea* were used in combination with molecular tools try and answer these questions. We used four genes, both nuclear and mitochondrial, to test the hypothesis that *H. caerulea* was introduced to Palmyra from the Caribbean: 18s rDNA, mtDNA *rnl* (large subunit rDNA) and the standard barcoding fragment; cytochrome oxidase subunit 1 (CO1) along with the suggested extension, which is more variable and thought to provide greater resolution for intraspecific differences (Rot et al., 2006).

## Methods

### Study species

**Figure 1:**
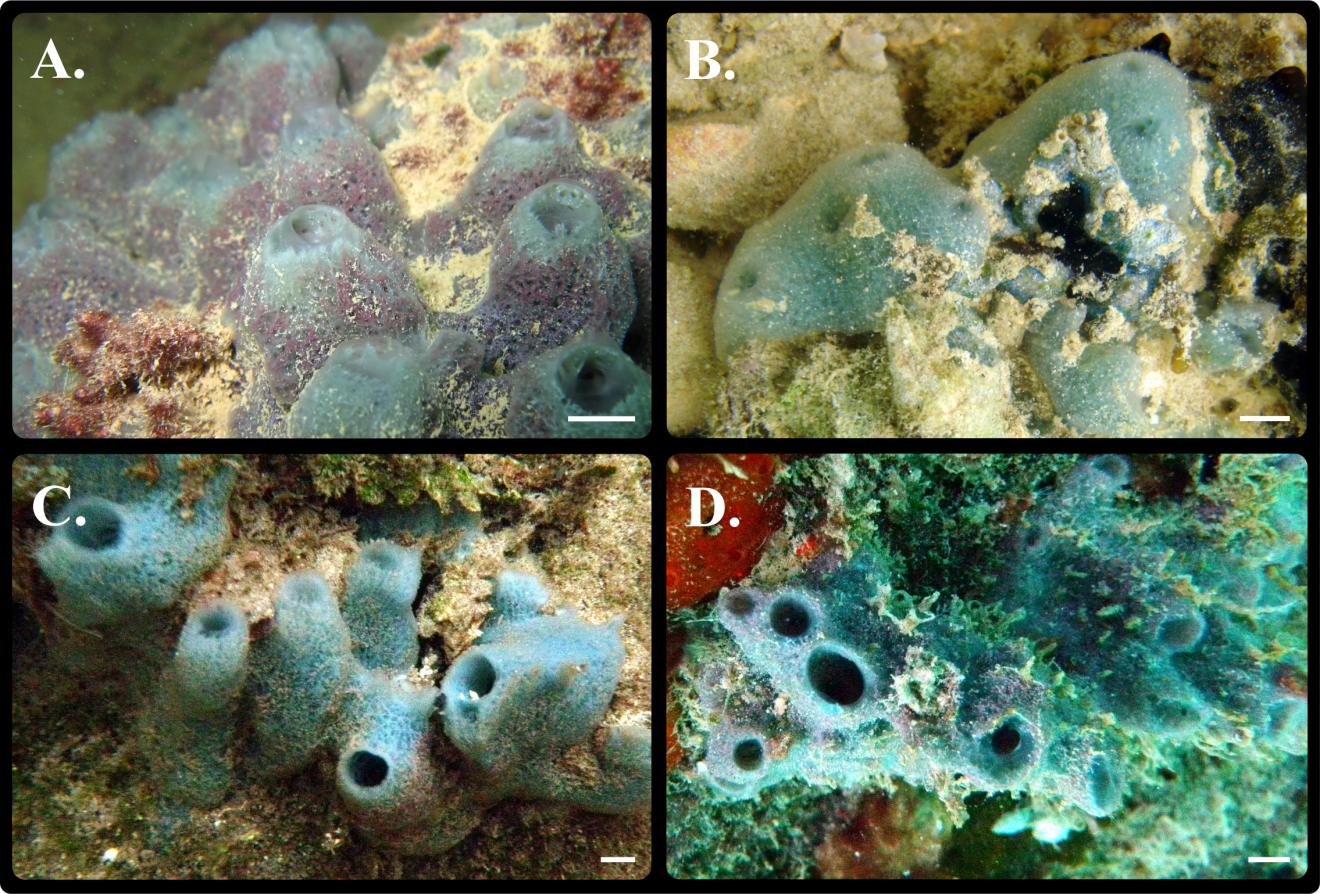
Photos of sponge morphs. *In-situ* images of *Haliclona (Soestella) caerulea* samples collected from (A) Palmyra Atoll (B) Hawaiʻi (C) Caribbean (St John) (blue morph) (D) Caribbean (St Thomas) (purple morph). Scale bars represent 1 cm.

*Haliclona* (*Soestella*) *caerulea* (Hechtel, 1965) (family Chalinidae Gray, 1867), commonly known as the ‘Blue Caribbean sponge,’ is found on rocks, dock pilings and mangrove roots in shallow water embayments, harbours and disturbed habitats with restricted water flow (Hechtel, 1965; Cubit & Williams, 1983; Wulff, 1997b; De Weerdt, 2000; DeFelice, Eldredge & Carlton, 2001). Synonyms of this species include: *Sigmadocia caerulea*, *Haliclona caerulea and Haliclona* (*Gellius*) *caerulea*, but *Haliclona* (*Soestella*) *caerulea* is the currently accepted name in the World Register of Marine Species (WoRMS). *Haliclona (Soestella) caerulea* is characteristically blue, but the colour can be purple when in association with the red branching coralline alga *Jania adherens* (Lamouroux, 1816) (Fig. 1), a mutualistic relationship often seen in populations from the Pacific Coast of Panama (Wulff, 1997a), Mexico (Carballo & Avila, 2004) and Palmyra Atoll (Knapp et al., 2011). The megasclere spicules are bent oxeas (82–230 µm), and the microscleres are C-shaped sigmas (18–30 µm) (Hechtel, 1965; DeFelice, Eldredge & Carlton, 2001; Carballo et al., 2006; Cruz-Barraza & Carballo, 2008), although these latter structures are less common in the purple mutualistic forms (Hechtel, 1965).

### Sampling design

**Figure 2:**
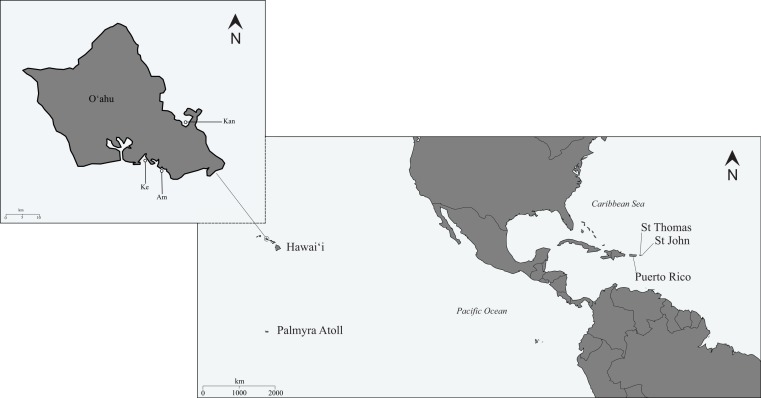
Map of sampling locations. Map of the sampling locations for *Haliclona (Soestella) caerulea* in the Caribbean and Pacific along with the individual collection sites on Oʻahu, Hawaiʻi: Keʻehi harbour (Ke), Ala Moana (Am) and Kāneʻohe Bay (Kan).

Between 2008 and 2009, a total of 313 specimens were sampled from 7 locations in the Line Islands, Hawaiʻi and the Caribbean. Samples were collected on SCUBA from Palmyra Atoll (in the Line Islands), and on snorkel in Hawaiʻi and the Caribbean (Fig. 2 and Table 1), and span a sampling range of approximately 10,400 km. All samples from Palmyra Atoll were collected under the USFWS special use permits 12533-08004 and 12533-09014. To try and mitigate *in-situ* misidentification, where possible, the purple morph was sampled as the association of *H. caerulea* with *J. adherens* is meant to be one of the distinguishing features of this species (Wulff, 1996). This association is also related to depth (Enríquez, Ávila & Carballo, 2009) so where possible deeper sites were sought to find the more distinguishable purple morph. In Hawaiʻi the predominant morph was blue, in Palmyra it was purple and in the Caribbean it was more variable (Fig. 1). Due to the high proportion of *J. adherens* in the purple morphs, the tissue fragments were taken from the base of the sponge where there is more blue sponge tissue compared to the associated algae. On average, 30–60 samples were collected from each location with the exception of Palmyra Atoll where 100 individuals were collected and Keʻehi boat harbour where only 10 specimens were collected (representing all individuals found). To avoid sampling clones, individuals were collected at least 2 m apart from each other and stored in individual 2 ml Eppendorf tubes. Immediately after collection, to reduce specimen atrophy, the seawater was replaced with saturated salt DMSO (dimethyl sulfoxide) buffer for storage, a highly efficient DNA preservation solution for invertebrates particularly in remote field locations (Dawson, Raskoff & Jacobs, 1998; Gaither et al., 2011).

**Table 1:** Site info. Location, code IDs and numbers of sequences included in the final analyses of ‘*Haliclona (Soestella) caerulea*’ samples.

Sites	Code	Region	Site description	Depth (m)	Latitude & Longitude	*n*
Ala Moana	(Am)	Hawaiʻi	harbour, sandy rocky substrate	≈2	21°17′N, 157°50′W	7
Keʻehi Bay	(Ke)	Hawaiʻi	harbour, pillings	≈2	21°19′N, 157°53′W	6
Kāneʻohe Bay	(Kan)	Hawaiʻi	backreef, sandy rocky substrate	≈2	21°25′N, 157°47′W	13
St John Island	(StJ)	Caribbean	mangrove roots	≈3	18°19′N, 64°44′W	7
St Thomas Island	(StT)	Caribbean	mangrove roots	≈3	18°18′N, 64°54′W	3
Puerto Rico	(PR)	Caribbean	mangrove roots	≈3	18°17′N, 67°12′W	5
Palmyra Atoll	(PA)	Line Islands	lagoons, sandy rocky substrate	≈8	05°53′N, 162°5′W	15
**Total**						**56**

### Spicule analyses

To identify any local site level morphological differences between specimens the oxea lengths from all locations (see Fig. 2) were measured using ImageJ (Abràmoff, Magalhães & Ram, 2004) based on photographs taken under a light microscope. To prepare the samples, a 2 mm^3^ fragment was taken from each specimen and placed in a 1 ml Eppendorf tube filled with sodium hypochlorite to remove the tissue and leave only the silicate spicules. After 24 h the samples were rinsed with distilled water three times. The samples were then vortexed and a drop of water containing spicules was pipetted onto a slide, with a cover slip and a drop of oil. Thirty to forty spicules were photographed under the microscope with the thirty clearest photos measured. A non-metric multidimensional scaling (nMDS) ordination (Anderson, 2001) was produced, based on a normalized Euclidean distance matrix, to visualize the variation in sample spicule lengths across sites. The nMDS ordination was created using PRIMER v6 (Clarke & Gorley, 2006) with the PERMANOVA+ add-on (Anderson, Gorley & Clarke, 2008).

### DNA extraction and PCR amplification

Individual sponge samples were extracted using a Qiagen DNeasy Tissue Kit following the manufacturer protocol. After initial thorough screening with 21 primer sets including those from Jarman, Ward & Elliott (2002) and Redmond et al. (2007), four regions were successfully sequenced and all samples were screened with these primers. The primer regions included three mitochondrial regions: CO1 and CO1 extension (ext.) (mtDNA) and *rnl* (rDNA) and the nuclear region 18s (rDNA) (see Table 2). CO1 is one of the three protein coding subunits in the cytochrome c oxidase complex involved in aerobic metabolism, *rnl* is a large ribosomal subunit in the metazoan mitochondrial DNA (Boore, 1999), and 18s is a highly conserved nuclear eukaryotic ribosomal DNA gene.

**Table 2:** Primer info. Primers used in the PCR and sequencing of ‘*Haliclona (Soestella) caerulea*’ samples.

Primer name	Region	Primer sequence (5′–3′)	Reference
dgLCO1490	CO1	GGT CAA CAA ATC ATA AAG AYA TYG G	Meyer, Geller & Paulay (2005)
dgHCO2198		TAA ACT TCA GGG TGA CCA AAR AAY CA	
COX1-R1	CO1 ext.	TGT TGR GGG AAA AAR GTT AAA TT	Rot et al. (2006)
COX1-D2		AAT ACT GCT TTT TTT GAT CCT GCC GG	
diplo-rnl-f1	*rnl*	TCG ACT GTT TAC CAA AAA CAT AGC	Lavrov, Wang & Kelly (2008)
diplo-rnl-r1		AAT TCA ACA TCG AGG TSG GAA AC	
18s_1-600_F	18s	GCC AGT AGT CAT ATG CTT GTC TCA	This paper^a^
18s_1-600_R		GAC TTG CCC TCC AAT TGT TC	

**Notes.**

a Modified from Redmond et al. (2007).

Standard universal barcoding primers for the CO1 region from Folmer et al. (1994) failed to consistently amplify a product via PCR, therefore we utilized the degenerate primers developed by Meyer, Geller & Paulay (2005). Due to the lack of variability in the standard CO1 region in sponges (Duran & Rützler, 2006; Wörheide, 2006), we sequenced the suggested downstream extension region described by Rot et al. (2006), which includes an intron and has higher substitution rates (Erpenbeck, Hooper & Wörheide, 2005). After alignment and trimming, the final sequence lengths were 554 and 318 base pairs (bp) for CO1 and CO1 ext., respectively. The Porifera-optimized primers diplo-*rnl*-f1 and diplo-*rnl*-r1 described by Lavrov, Wang & Kelly (2008) were used to amplify the mitochondrial *rnl* ribosomal RNA region (≈700 bp), of which 311 bp were examined in this study. The 18s rDNA region is approximately 1,800 nucleotides long in Haplosclerida sponges (Redmond & McCormack, 2008) but we included only the first 407 bp after multiple sequence alignment and trimming.

PCR amplifications were performed in 15 µl reactions consisting of: 8.5 µl nanopure water, 1.5 µl 10 × NH_4_ buffer, 0.45 µl MgCl_2_, 0.30 µl dNTP’s (10 mM total), 2.0 µl 40× bovine serum albumin (BSA), 0.1 µl *Taq* polymerase (Biolase, Bioline), 0.075 µl of each primer (10 µM) and 2 µl of DNA template (∼20 ng/ µl) with the following thermal cycler profile: 95 °C for 3 min, followed by 35 cycles at 94 °C for 30 s, 58 °C (*rnl* and CO1) or 50 °C (18s) for 45 s, 72 °C for 45 s and a final extension step at 72 °C for 10 min. The PCR products were then purified using 1.125 µl Exo-FAP (final composition: 0.75 units Exonuclease I and 0.5 units FAST alkaline phosphatase (FastAP), Thermo Fisher Scientific, Waltham, Massachusetts, USA) per 7.5 µl of PCR product incubated at 37 °C for 60 min and then deactivated at 85 °C for 10 min. The purified samples were then sequenced using BigDye Terminators (PerkinElmer) at the EPSCoR core genetics facility at the Hawaiʻi Institute of Marine Biology on an ABI-3130XL automated sequencer.

### Data analysis

All sequences were evaluated and aligned using Geneious Pro (v.5.3.6) (Drummond et al., 2011) and all GenBank accession numbers are supplied in Appendix S1. The Poriferan origin of the sequences was confirmed using the BLAST search engine in GenBank and BOLD Systems (Barcode of Life Data). For all data-sets, the outgroup taxa was *Amphimedon queenslandica*, which is also from the order Haplosclerida (Van Soest & Hooper, 2002) and currently the only whole sponge genome (Srivastava et al., 2010), therefore there was sequence data for this species for all the loci included in this study.

If, after numerous sequencing attempts, samples did not produce comparable sequences across all regions (CO1, CO1 ext. *rnl* and 18s), they were removed from the analyses. Simple neighbour joining (NJ) trees (in Mega5 (Tamura et al., 2011) with a p-distance model, pairwise deletion of gap positions and 1,000 bootstrap replicates) were generated to provide an overview of the data for each region. The number of included samples was then reduced to improve the visualization of the results and reduce analysis time while still representing the genetic diversity between samples with up to 5 identical individuals selected per tree grouping per region resulting in 56 samples across all locations (see Table 1).

To determine the best-fit nucleotide substitution model for each data set, corrected Akaike information criterion (AICc) model selections were performed by jModelTest v2.0 (Posada & Crandall, 1998). The jModelTest results identified Hasegawa, Kishino and Yano HKY + G (gamma) models for both CO1 regions and HKY for the *rnl* region as the best fitting prior models of evolution. Transversion (TVM + G) and transitional models (TIM2ef + G) were selected for the concatenated mitochondrial and nuclear data sets, respectively. However, for the Bayesian and maximum likelihood analyses, TVM and TIM models were not available in MrBayes and MEGA5, therefore a general time reversible model with gamma rates (GTR + G) was used as the closest available model (Posada & Crandall, 2001; Zakharov et al., 2009).

The mitochondrial regions (CO1, CO1 ext. and *rnl*) for all 56 samples were examined separately to determine if there were major differences in tree topologies among gene trees. Results indicated that there were no major differences in topology (see Appendix S2); therefore, the mitochondrial regions were additionally concatenated to form a total evidence tree for the mtDNA to increase the resolution. To determine within and between species level differences among the subclades, the pairwise percent sequence divergence (P) (see Table 3) was calculated for all gene regions in MEGA5 as the proportion of variable nucleotide sites in each alignment.

**Table 3:** Pairwise values. Total number of base pairs (bp), samples (n), haplotypes (H) and pairwise percent sequence divergence per site, relative to all other sites (P) for each sampling location and across all *Haliclona (Soestella) caerulea* samples.

Sites	Gene	Total length (bp)	*n*	H	P (%)
Palmyra Atoll	CO1	554	15	3	27
	CO1 ext.	318	15	2	11
	*rnl*	366	15	1	0
	mtDNA	1,182	15	4	16
	nDNA	407	15	3	11
Hawaiʻi	CO1	554	26	3	31
	CO1 ext.	318	26	3	15
	*rnl*	366	26	3	27
	mtDNA	1182	26	6	27
	nDNA	407	26	3	13
Caribbean	CO1	554	15	2	20
	CO1 ext.	318	15	2	19
	*rnl*	366	15	2	22
	mtDNA	1182	15	3	21
	nDNA	407	15	3	10
**All sequences**	CO1	554	56	5	32
	CO1 ext.	318	56	6	27
	*rnl*	366	56	3	27
	mtDNA	1182	56	12	31
	nDNA	407	56	6	14

**Notes.**

ext. extension region

**Figure 3:**
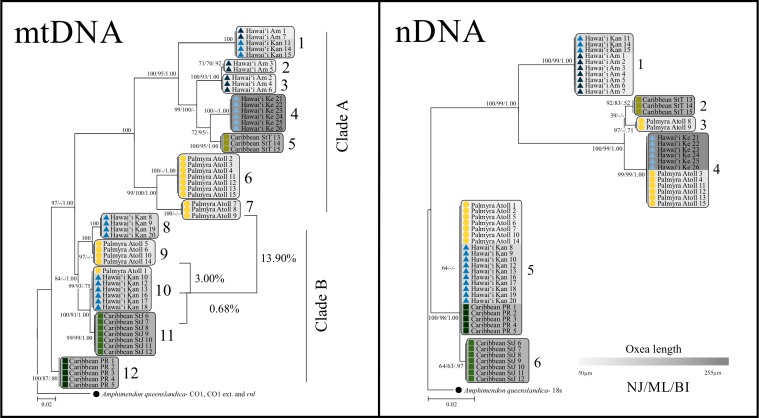
mtDNA and nDNA trees. Neighbour joining phylograms of the 56 sequenced ‘*Haliclona (Soestella) caerulea*’ samples along with the outgroup species *Amphimedon queenslandica* with the concatenated total evidence mtDNA tree and the 18s nDNA trees with branch support from Neighbour Joining (NJ), Maximum Likelihood (ML) and Bayesian Inference (BI) analyses. Regional names (Hawaiʻi, Palmyra and Caribbean) are followed by site names: Keʻehi harbour (Ke), Ala Moana (Am) and Kāneʻohe Bay (Kan), St John Island (StJ), St Thomas Island (StT) and Puerto Rico (PR) and then the sample number per region. The scale bar is based from the average oxea lengths (Appendix S2) for each sample and overlaid on the mtDNA and nDNA trees. The two clades A and B are further divided into subclades, which are numbered separately for each tree. Support values indicated as 100 represent identical support from each analysis (i.e., 100/100/1.0). Scale bar = 0.02 substitutions per 100 sites. % values represent the lowest sequence divergence values between subclades and clades.

Phylograms were created separately for each gene region (CO1, CO1 ext., *rnl* and 18s) as well as for the entire concatenated mitochondrial region (Fig. 3 and Appendix S2) with NJ, maximum likelihood (MP) and Bayesian inference (BI) values supporting branch topologies. Maximum likelihood trees were generated by MEGA5 using the nearest-neighbour interchange (NNI) heuristic method with gaps included at all sites and 1,000 bootstrap replicates. Neighbour joining trees were also constructed using MEGA5 with the same conditions as described above. Bayesian analyses were conducted in MrBayes v3.1.2 (Ronquist & Huelsenbeck, 2003) using the Markov chain Monte Carlo (MCMC) analyses (Geyer, 1991) for 300,000 generations sampling 4 chains every 300 generations with the first 250 trees (75,000 generations) discarded at the burn-in period. All trees were based on the NJ topology and drawn in MEGA5.

One sequence of the CO1 region (no extension) for *H. coerulea* (*sic*) from the Caribbean (ID# EF519619) (Erpenbeck et al., 2007) was sourced from GenBank and included in our analyses to confirm the identification of *H. caerulea* based on the sponge barcoding basic region.

## Results

### Morphological characteristics

**Figure 4:**
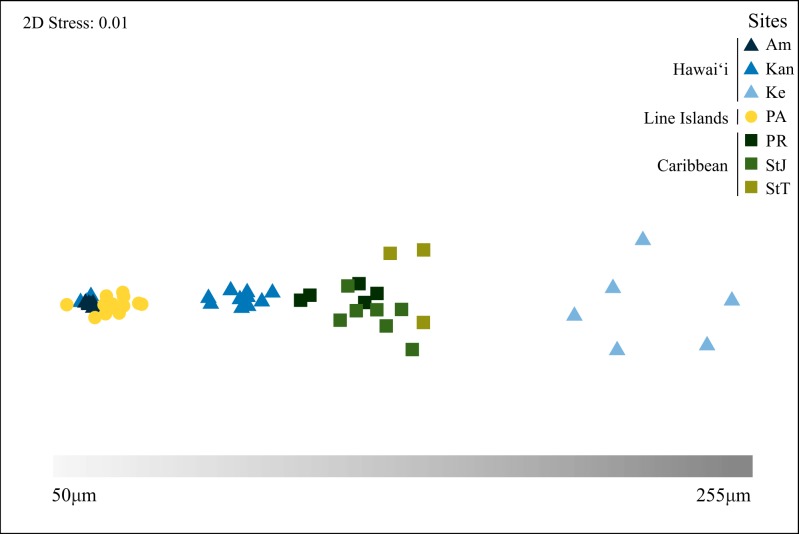
nMDS of spicules. Non-metric multidimensional scaling (nMDS) ordination visualizing grouped *Haliclona (Soestella) caerulea* oxea lengths (µm) across sites Keʻehi harbour (Ke), Ala Moana (Am) and Kāneʻohe Bay (Kan) (Hawaiʻi), Palmyra Atoll (PA) (Line Islands) and Puerto Rico (PR), St John Island (StJ), St Thomas Island (StT) (Caribbean) based upon a normalised Euclidean distance matrix. The scale bar is based from the average oxea lengths (Appendix S2) for each sample.

The spicule lengths for all samples ranged from 50 µm to 290 µm (Table 4). Keʻehi harbour had the largest range out of all the locations with oxeas measuring from 155 to 290 µm, whilst all others ranged from 50 to 209 µm as visualized on the nMDS (Fig. 4). Each point on the nMDS indicates how similar or dissimilar each sample is from every other based on oxea lengths, so the closer two points are the more similar their oxea lengths. All Caribbean and Palmyra Atoll samples grouped relative to their geographic location. Samples from Kāneʻohe Bay and Ala Moana in Hawaiʻi, group with those from Palmyra Atoll and the Caribbean, whilst the Keʻehi harbour samples cluster on their own. Appendix S3 also reveals no clear separation based on clades, with the exception of those from Keʻehi harbour.

**Table 4:** Spicule mean, min, max. ‘*Haliclona (Soestella) caerulea*’ mean and standard error (SE), minimum (Min) and maximum (Max) oxea lengths (µm) from: Palmyra Atoll (PA), Ala Moana (AM), Kāneʻohe Bay (Kan), Keʻehi harbour (Ke), Puerto Rico (PR), St John Island (StJ) and St Thomas Island (StT) based on Appendix S2 and the two clades as indicated in Fig. 3.

Location	Site Code	Mean ± SE	Min	Max
Hawaiʻi	Am	59.5 ± 0.2	50.5	68.3
Hawaiʻi	Kan	94.4 ± 1.1	51.0	127.5
Hawaiʻi	Ke	230.0 ± 2.0	155.4	290.5
Palmyra Atoll	PA	106.1 ± 0.4	44.9	83.2
Caribbean	PR	136.2 ± 1.1	103.5	167.9
Caribbean	StJ	146.3 ± 1.1	109.2	199.1
Caribbean	StT	158.1 ± 2.3	111.0	208.9
Clade				
A	n/a	92.47 ± 2.39	44.9	290.5
B	n/a	114.3 ± 1.09	45.3	199.1

The average spicule lengths calculated from Appendix S4 when overlaid on the nMDS and topographical trees (Fig. 3) revealed polyphyletic groups that were not consistent with the molecular data, including within subclades, with the exception of subclade 4 in the mtDNA tree, which confirmed that the Keʻehi harbour individuals were significantly different from all other locations. In addition, we found no consistency with colour morph and genetic groups; for example all the samples collected from Palmyra Atoll were purple morphs and they span both clades and subclades including subclade 10, which is also made up of blue Hawaiian samples.

### Individual gene regions

Contrary to the expectation of a monophyletic species tree, our data revealed two deeply divergent clades. When examining each of the CO1, *rnl* and 18s regions (excluding the CO1 extension) individually, two divergent clades were evident with little differentiation in clade B (Fig. 3 and Appendix S2). The CO1 region identified five subclades; with the GenBank sequence from Erpenbeck et al. (2007) nesting within clade B, which includes samples from all sites except St Thomas. In BLAST and BOLD searches samples clustering within clade A were found to be most similar to *Halichondria* spp. (order Halichondrida) with ≤91.7% pairwise identity (PI) for all subclades whereas samples from clade B were most similar to Haplosclerida species with 100% (PI). The only exception was subclade one in clade A, which was most similar to a Callyspongia sp. (with 95.3% pairwise identity), which is also a Haplosclerida species like those in clade B. Further resolution within clade B was not possible with the CO1 marker or the *rnl* region because all sequences were identical. The CO1 ext. resolved clade B more than any other individual marker resulting in 6 subclades in total (compared to 5 for the regular COI region) (Appendix S2).

The nuclear 18s marker was largely congruent with the mitochondrial data (Fig. 3). Although samples from St John form a subclade (group 6), they are not well supported by the NJ or ML analyses, and when looking between samples there is only 1 bp (0.3% sequence divergence) difference between groups 5 and 6. Thus, while the 18s nDNA region shows essentially similar patterns to the CO1 and *rnl* gene trees, supporting a single Caribbean group, this was in sharp contrast to the CO1 ext. locus.

### Concatenated mtDNA versus nDNA

As with the individual gene trees, there were no major discrepancies between the concatenated mtDNA and nDNA regions (Fig. 3), with each supporting two major clades A and B subdivided into a number of different terminal subgroups, all supported by congruent significant values from the NJ, ML and BI analyses. The concatenated mtDNA tree was the best resolved with the CO1 ext. providing most of the polymorphism. Still, this region is short and did not resolve all subclades (see Appendix S2 and Fig. 3). Overall, the concatenated mtDNA tree revealed genetic structure at several levels, and highlights that there are geographic differences between the samples. Although tree topology was congruent, mtDNA revealed roughly 3-fold greater sequence divergence among sites on average (Table 3), revealing slower rates of sequence evolution in the nDNA (∼14% pairwise divergence) than the mtDNA (∼31% pairwise divergence) marker.

In the mtDNA tree clade B had 5 subclades and the only subclade in the mtDNA tree with samples from more than one geographic region was subclade 10 in clade B, which contained both Palmyra and Hawaiian samples. The next closest subclade (11) grouped Caribbean samples from St John Island, which had 0.68% sequence divergence (8 bp different, all from the CO1 ext. region) compared to subclade 10. The remaining subclades 8, 9 and 12 in clade B had sequence divergence values of 4.0, 3.0 and 4.9%, respectively, when compared to subclade 10. Overall, comparing all subclades in clade B, the pairwise sequence divergence was 7.5%, compared to 22.8% among all samples in clade A. Neither the clades nor individual subclades could be identified further because voucher samples with accessioned sequences in the GenBank or BOLD Systems (Barcode of Life Data) database are currently not comprehensive enough to provide confident identification.

## Discussion

Cryptic species can be a major conundrum when trying to discern species introductions (Bickford et al., 2007) and can be particularly difficult when working with sponges, which are often enigmatic and difficult to identify accurately in the field (Wulff, 2001; Wörheide, Erpenbeck & Menke, 2007). Even among sponges, Haplosclerids in particular are considered a taxonomically difficult order because of the low variability in morphological characteristics, coupled with high levels of macromorphological plasticity; recent phylogenetic investigations suggests that this order needs revising at all taxonomic levels (Redmond et al., 2011; Cárdenas, Pérez & Boury-Esnault, 2012; Redmond et al., 2013). In particular, examining species level differences in this order is fraught with difficulties including cryptic species, lack of fixed morphological differences and problems defining within, as opposed to, between species level variation in both morphological and molecular traits (Raleigh et al., 2007; Redmond & McCormack, 2008; DeBiasse & Hellberg, 2015). Establishing the level of intra and interspecific differences between locations is critical in determining whether a species is introduced (one species spread across several locations) or endemic (multiple species in their own locations) (Xavier et al., 2009; Bastos et al., 2011). Therefore, confirming both the species identification and the relationship of the species to populations elsewhere is fundamental to any management actions. This is particularly true for potential introductions of understudied groups with little taxonomic resolution and no baseline biodiversity surveys at remote locations, such as is the case with *Haliclona (Soestella) caerulea* which is found in the lagoons of the isolated, and otherwise, near-pristine Palmyra Atoll (Knapp et al., 2011).

We present molecular evidence that *H. caerulea* is found at Palmyra Atoll, Hawaiʻi and the Caribbean; however, taxonomic uncertainty complicates the phylogeographic relationships between sampling regions. Overall, three of the four gene regions (CO1, rnl and 18s) provided low resolution between the samples and broadly grouped individuals into potentially two order level clades (cf. Halichondrida (Gray, 1867) and Haplosclerida (Topsent, 1928)) (Appendix S2 and Fig. 3) with almost no sequence divergence within clade B. The lack of clade B sequence divergence within this diverse group leaves questions of whether this is a cryptic species complex or the available genetic markers lack the resolution to resolve species in this clade, a problem reminiscent of that in corals (e.g., Romano & Palumbi, 1996; Forsman et al., 2010; Swierts et al., 2013). Overlaying the oxea spicule lengths onto the phylogenetic tree did not improve data resolution between the clades. However, the CO1 ext. and the concatenated mtDNA trees did reveal some evidence for cryptic species, with up to twelve subclades, each corresponding to a single geographic location with the exception of one subclade (Fig. 3). Despite the lack of taxonomic resolution in clade B, geographic restriction of subclades suggests some taxonomic resolution and provides scope to identify haplotypes that occur far outside the geographic range occupied by other subclades (Fig. 3). The sequence divergence among these geographic regions provides a measurement against which to compare putative introduced populations in Hawaiʻi and at Palmyra. The close relationship of St John samples to the Hawaiʻi and Palmyra ones, and the extremely low levels of sequence divergence across markers between those sites, is consistent with the hypothesis of introduction of *H. caerulea* to Palmyra via human transportation (see Fig. 3).

### Using morphological characteristics to identify *H. caerulea*

The spicule lengths grouped by the nMDS ordination (Fig. 4) were not congruent with any gene tree topologies (Fig. 3), and did little to help explain the variability between groups with the exception of the Keʻehi harbour samples, which fell into clade A and were distinctly longer (155–290 µm) than all other locations (Table 4). Therefore, it would appear that it might be possible to exclude some samples based on oxea length. Unfortunately the lower range of spicule lengths still falls within the documented size range for *H.caerulea*, but the maximum size does exceed this range therefore in the future it may be possible to at least exclude some samples based on spicule maximum length.

Grouping the spicules by clade (Table 4 and Appendix S3) revealed a large overlap in oxea lengths with clade A samples ranging from 45 to 199 µm and the clade B samples between 45 and 290 µm revealing a large overlap in oxea lengths. Globally, oxea lengths for *H. caerulea* range from approximately 82–230 µm and vary with location and water flow rates (Carballo et al., 2006) for example in Hawaiʻi they range from 170 to 230 µm long (DeFelice, Eldredge & Carlton, 2001), in Mexico 82–210 µm (Cruz-Barraza & Carballo, 2008) and in Jamaica, the site of the original description, 117–200 µm long (Hechtel, 1965). Despite the lack of resolution between clades in our data, there does appear to be a slight geographical trend (Fig. 4) with spicule lengths getting smaller from East to West (in clade B). This relationship with spicule length is also evident in Callyspongia, another Haplosclerida genus, in the Caribbean (DeBiasse & Hellberg, 2015), where oxea lengths are concordant with geographical region and not genetic differentiation. Together, these observations suggest that the use of spicule lengths alone are not a suitable tool for identification of Haplosclerida species because it is either highly dependent on environmental conditions, has homoplastic non-distinct morphological features or both. However, the addition of other micromorphological measurements (e.g., sigma spicule lengths) and spongin architecture may provide further differentiation in the future.

Unfortunately, spicule lengths overall may not even be useful to identify sponges at even the order level as some Halichondrida species have similar oxeas to those found in the Haplosclerida (Erpenbeck & Soest, 2002), which explains the lack of morphological resolution found in this study. It is also interesting to note that subclade 1 (clade A) (Fig. 3) when blasted against GenBank and BOLD was most similar to Haplosclerida spp. like those in clade B supporting the notion that the current databases are not currently comprehensive enough to provide confident identification and/or that morphological classification of sponges even at the order level is still disparate with the genetic data. In contrast to the micromorphological traits, the genetic analyses resolve the differences between the two clades distinctly and reliably, and the concatenated mtDNA tree resolves subclades that may be either geographic or taxonomic in nature as outlined below.

### Gene loci

We found considerable genetic diversity among samples morphologically identified as *H. caerulea.* Overall, the two mtDNA markers: CO1 and *rnl* provided the lowest resolution. Mitochondrial genes have many advantages for phylogeographic and phylogenetic studies (Avise, 2000; Lavrov, 2007), and the sponge barcoding project uses the CO1 region as its basic identification region (Wörheide, Erpenbeck & Menke, 2007). COI has been used to successfully resolve some sponges down to species level (e.g., Pöppe et al., 2010). However, often in sponges (and some other non-bilaterians such as Cnidarians) the accumulation of mutations in mitochondrial genes is too slow to determine population structure or even species differences (Shearer et al., 2002; Duran, Pascual & Turon, 2004; Wörheide, 2006; Huang et al., 2008). The low resolution in our *rnl* and CO1 trees highlight this issue, and supports the recommended inclusion of a sponge barcode extension region to aid in the differentiation between sponge species (Rot et al., 2006). While this extension clearly improved resolution among our geographic samples, the best resolution was provided by the concatenated mtDNA tree.

The 18s rDNA is slowly evolving and frequently used to resolve higher taxonomic relationships (e.g., Peterson & Addis, 2000; Borchiellini et al., 2001) making it generally unsuitable for resolving finer population structure (Wörheide et al., 2002; Wörheide, Hooper & Degnan, 2002); however, it was the only nuclear locus that consistently amplified among all samples for this study. When looking at the number of variable sites in *H. caerulea*, the sequence divergence in the mtDNA was approximately twice that of the 18s nDNA sequences. Lavrov et al. (2005) made a similar observation and found sequence divergence to be 4.3 times greater in mtDNA compared to nDNA in the sponges *Geodia neptuni* (Sollas, 1886) and *Tethya actinia* (De Laubenfels, 1950a). Despite two-fold greater variation in the mtDNA, the sequence polymorphism is still too low in most cases to resolve species level differences (Hudson, Kreitman & Aguadé, 1987; Shearer et al., 2002; Duran, Pascual & Turon, 2004); however, the relatively high levels of polymorphism in the CO1 ext. offer a stark contrast. In the future, to further resolve this issue of cryptic speciation and this species introduction, it would desirable to include additional markers (e.g., Rua, Zilberberg & Solé-Cava, 2011) or even apply next generation RAD-seq techniques (e.g., Toonen et al., 2013) for these non-model organisms.

### Cryptic species within *H. caerulea*

The genetic data indicates possible cryptic taxa, which limits some interpretations of phylogeographical relationships. Despite morphological consistency across this broad geographic region, the CO1 data clearly indicate that this morphospecies is comprised of at least two groups. These two convergent groups each have large geographic distributions, because individuals from both clades were found to occur across all regions whereas the concatenated mitochondrial tree reveals that there appears to be 12 geographic subclades, which may be isolated populations or may represent further cryptic diversification.

Although we cannot completely rule out the possibility that *H. caerulea* is endemic but previously unrecognized at Palmyra, it seems exceedingly unlikely to observe such shallow genetic diversification across approximately 10,000 km. Given geographic restriction of other subclades and the extremely low dispersal potential of larvae of *H. caerulea* (Maldonado & Young, 1999) in the absence of human transportation (DeFelice, Eldredge & Carlton, 2001), it seems far more likely that *H. caerulea* has been transported to Palmyra Atoll from the Caribbean via Hawaiʻi. Unfortunately it is difficult to draw any clear conclusions on connectivity patterns between the sampling regions without more detailed knowledge of mutation rates, therefore it remains unknown whether *H. caerulea* was introduced just once or on multiple occasions. The sequence variation seen at Palmyra is exceptional in comparison to similar sampling effort at other sites. Given the extremely low rates of sequence divergent at mtDNA in sponges (Shearer et al., 2002; Duran, Pascual & Turon, 2004; Wörheide, 2006), this high variation is most likely to have come from multiple introductions of diverse populations or closely related species from unsampled geographic locations. Further geographic sampling throughout the Caribbean will be required to test this hypothesis in the future.

Focusing on the target clade B, which more closes matches Haplosclerida order species during BLAST searches, the concatenated mitochondrial tree indicates that the same species is found at Palmyra Atoll and Hawaiʻi with samples from both geographic regions, which are approximately 1,100 km apart, falling into subclade 10 with identical haplotypes over the 1,182 bp sampled. The Caribbean and Palmyra Atoll, which are approximately 10,000 km apart, are also very closely related with only 0.68% (8bp) divergence between groups 10 and 11. Similarly, Duran, Pascual & Turon (2004) found low nucleotide diversity (0.0006) in samples of *Crambe crambe* (Schmidt, 1862) spanning 3,000 km from the Western Mediterranean to the Atlantic. Furthermore, low sequence divergence has also been shown in *Astrosclera willeyana* (Lister, 1900) across a 20,000 km range in the Indo-Pacific along with *Halisarca* spp. (Johnston, 1842) over 2,500 km across the Caribbean (Wörheide, 2006; Alvizu et al., 2013). Huang et al. (2008) found that intraspecific differences for sponges (taken from all CO1 sequences in GenBank in September 2006) averaged 0.60% (±0.10) compared to 3.76% (±0.57) for the closest congeneric interspecific distances. Based on that analysis, the percentage of divergence between groups 10 and 11 (0.68%) is consistent with these subclades being the same species. In contrast, the next closest subclades to the Palmyra-Hawaiian group, 8, 9 and 12 had divergence values of 4.0, 3.0 and 4.9%, respectively. These greater differences may indicate that samples in subclades 8 and 9 represent cryptic congeneric species-level differences. However, it is important to note that Huang et al. (2008) focused on sequences from the CO1 region in the analyses, most likely excluding the extension region identified by Rot et al. (2006), because the majority of sequence data from that study was collected before use of this marker became common. In our study, the CO1 ext. provided the greatest single locus resolution; without it, there was 0% sequence difference at COI among any clade B samples (Fig. 2). Given the higher divergence values in our concatenated tree are largely due to the CO1 ext., it remains unclear what the true species boundaries are between the subclades. When looking between clades, the sequence divergence between B (subclade 10) and A (subclade 7) is 13.9% in the concatenated tree, based on the mitochondrial sequences, which supports the premise that these are almost certainly not the same species. Clade A and B appear to represent higher level cryptic taxa (perhaps at the order level based on available information) that are presently all morphologically identified as *H. caerulea*.

Given the limited sampling effort, the high support values for each of these subclades, the geographic restriction of subclades, and number of substitutions between them, it seems most likely that there are greater rather than fewer species. Additional research is warranted to resolve whether these clades represent species or population level differences.

## Conclusions

We report surprising levels of genetic subdivision within the sponge *Haliclona* (*Soestella*) *caerulea* sampled from the native range in the Caribbean as well as suspected introduced populations in Hawaiʻi and at Palmyra Atoll in the Pacific Ocean. Our findings indicate morphologically indistinguishable individuals can be up to 13.9% divergent in their mtDNA haplotypes, and that there is considerable taxonomic uncertainty in this group because these divergent mtDNA clades appear to differ potentially at the order level but remain masked within this single morphospecies. Despite this taxonomic uncertainty, concatenated mitochondrial sequences identify up to 12 geographically restricted subclades that may represent population or species-level divergence. Albeit limited, this resolution provides a benchmark against which to compare samples from the Pacific and Caribbean oceans, and we find very low divergence (0.68%) between Palmyra and the Caribbean. In conjunction with the literature, the observed greater divergence among sites within the Caribbean than between Caribbean and Pacific locations supports the notion of a contemporary introduction, although alternative hypotheses cannot be ruled out. This research forms a foundation for further exploring these newly discovered cryptic species as their discovery will contribute towards understanding the evolution and conservation of this ecologically important group.

## Supplemental Information

10.7717/peerj.1170/supp-1 Appendix S1 GenBank Accession numbers Appendix S1—GenBank Accession numbers of cf. *Haliclona caerulea* sequences for all loci: CO1, CO1 ext., *rnl* and 18s, included in this study.

10.7717/peerj.1170/supp-2 Appendix S2 mtDNA trees **Appendix S2** Neighbour Joining phylograms of the 56 sequenced ‘*Haliclona (Soestella) caerulea*’ samples along with the outgroup species *Amphimedon queenslandica* with mitochondrial gene region CO1, CO1 extension (ext.) and *rnl*. with branch support from Neighbour Joining (NJ), Maximum Likelihood (ML) and Bayesian Inference (BI) analyses. Regional names (Hawaiʻi, Palmyra and Caribbean) are followed by site names: Keʻehi Bay (Ke), Ala Moana (Am) and Kāneʻohe Bay (Kan), St John Island (StJ), St Thomas Island (StT) and Puerto Rico (PR) and then the sample number per region. The two clades are divided into A and B with all subclades numbered separately for each tree. Support values indicated as 100 represent identical support from each analysis (i.e. 100/100/1.0). Scale bar = 0.02 substitutions per 100 sites.

10.7717/peerj.1170/supp-3 Appendix S3 Clade nMDS Appendix S2—Non-metric multidimensional scaling (nMDS) ordination based upon a zero-adjusted Bray-Curtis similarity matrix visualizing grouped *Haliclona (Soestella) caerulea* oxea lengths (µm) across clades A and B as defined by the concatenated mtDNA trees. The scale bar is based from the average oxea lengths (Appendix S2) for each sample

10.7717/peerj.1170/supp-4 Appendix S4 spicule measurements **Appendix 4** Oxea lengths (µm) (30 per sample), mean and standard error (SE), minimum (Min) and maximum (Max) measurements of the 56 sequenced ‘*Haliclona (Soestella) caerulea’* samples from sites: Palmyra Atoll (PA), Ala Moana (AM), Kāneʻohe Bay (Kan), Keʻehi harbor (Ke), Puerto Rico (PR), St John Island (StJ) and St Thomas Island (StT).

## Additional Information and Declarations

### Field Study Permissions

The following information was supplied relating to field study approvals (i.e., approving body and any reference numbers): This work was conducted under the US Fish and Wildlife Service (USFWS) special use permits (SUP) 12533-08004 and 12533-10002.

### DNA Deposition

The following information was supplied regarding the deposition of DNA sequences: GenBank Accession numbers supplied as Appendix S1.
