# Supplementary figures and images for: Cryptic species obscure introduction pathway of the blue Caribbean sponge (Haliclona (Soestella) caerulea), (order: Haplosclerida) to Palmyra Atoll, Central Pacific
Source: PeerJ. 2015 Aug 6;3:e1170. doi: 10.7717/peerj.1170 (PMC4558080; doi:10.7717/peerj.1170)

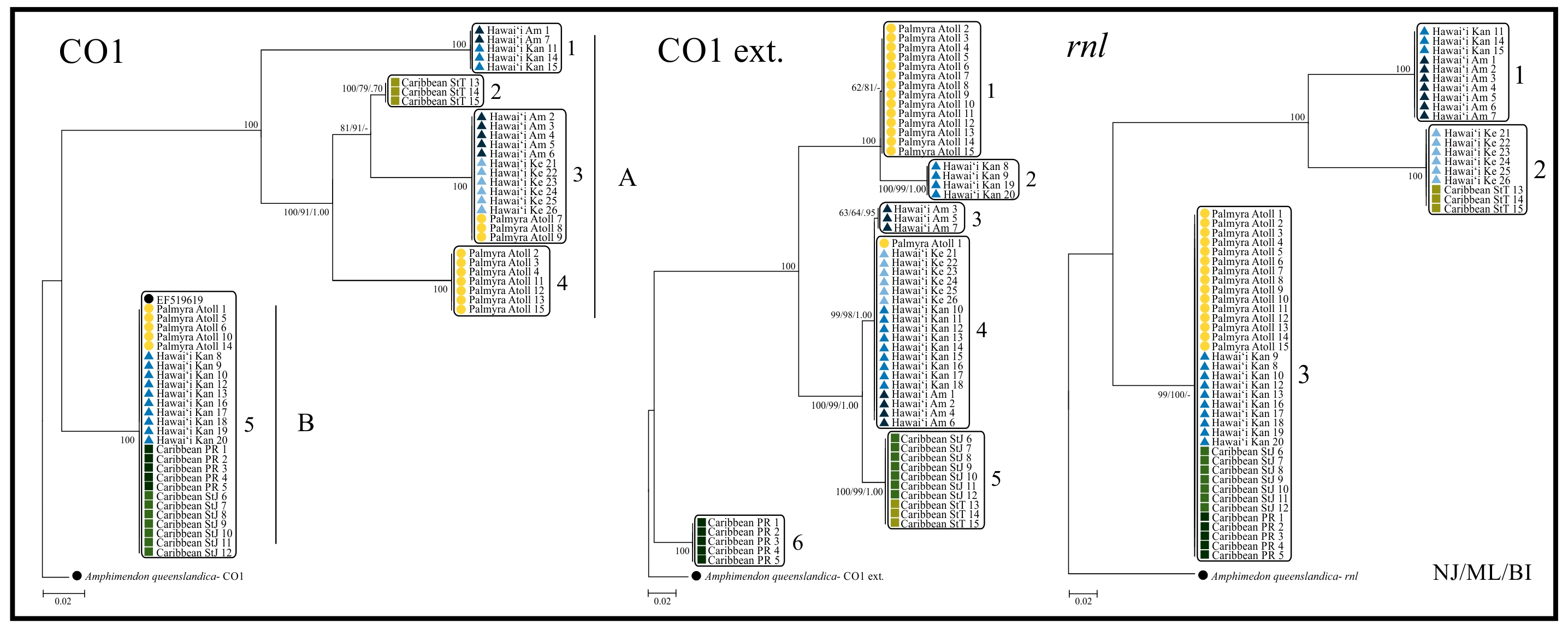

Supplement: Appendix S2 — Neighbour Joining phylograms of the 56 sequenced ‘Haliclona (Soestella) caerulea’ samples along with the outgroup species Amphimedon queenslandica with mitochondrial gene region CO1, CO1 extension (ext.) and rnl. with branch support from Neighbour Joining (NJ), Maximum Likelihood (ML) and Bayesian Inference (BI) analyses. Regional names (Hawaiʻi, Palmyra and Caribbean) are followed by site names: Keʻehi Bay (Ke), Ala Moana (Am) and Kāneʻohe Bay (Kan), St John Island (StJ), St Thomas Island (StT) and Puerto Rico (PR) and then the sample number per region. The two clades are divided into A and B with all subclades numbered separately for each tree. Support values indicated as 100 represent identical support from each analysis (i.e. 100/100/1.0). Scale bar = 0.02 substitutions per 100 sites. [file peerj-03-1170-s002.jpg]

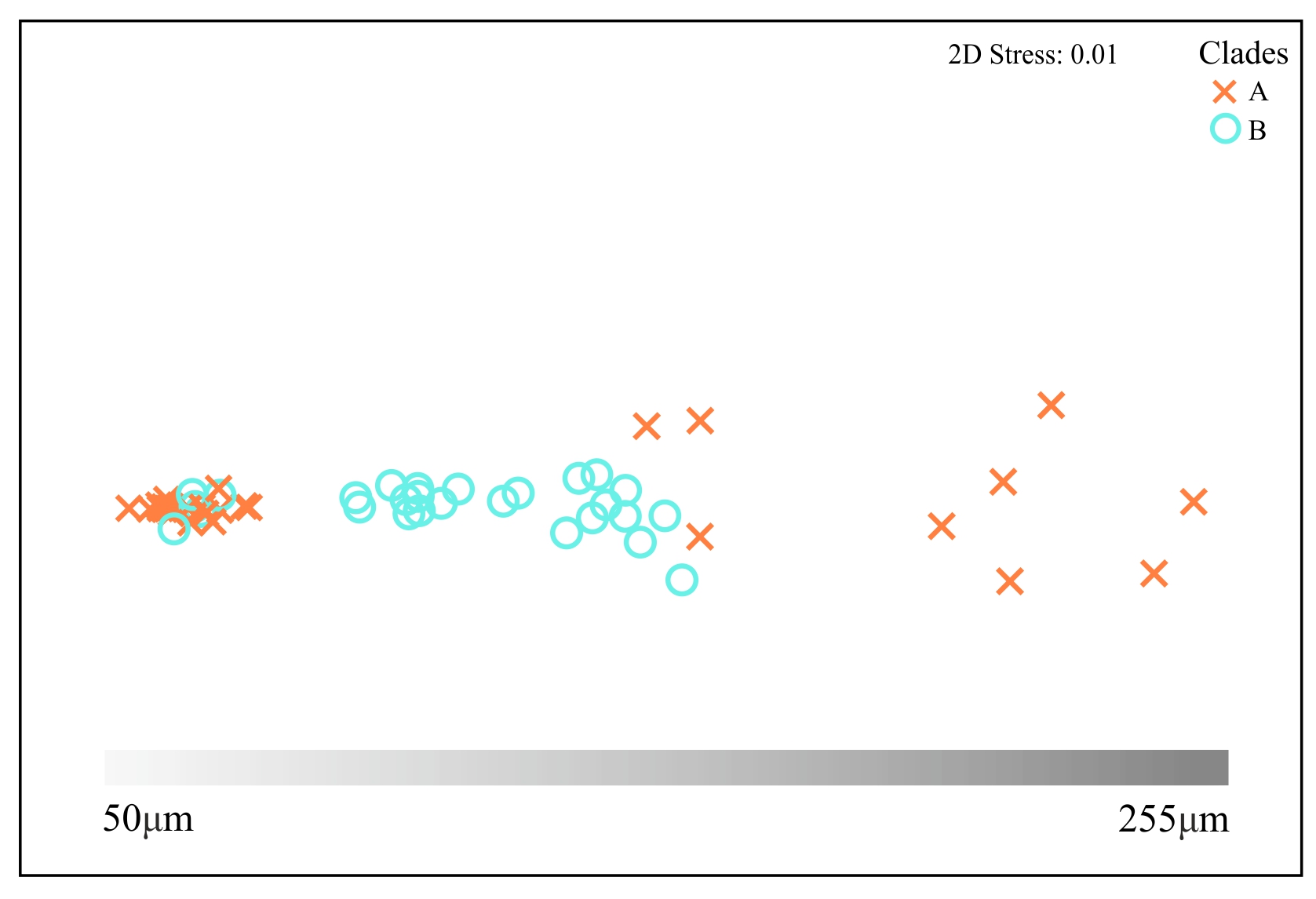

Supplement: Appendix S3 — Appendix S2—Non-metric multidimensional scaling (nMDS) ordination based upon a zero-adjusted Bray-Curtis similarity matrix visualizing grouped Haliclona (Soestella) caerulea oxea lengths (µm) across clades A and B as defined by the concatenated mtDNA trees. The scale bar is based from the average oxea lengths (Appendix S2) for each sample [file peerj-03-1170-s003.jpg]
